# Gender and Media Representations: A Review of the Literature on Gender Stereotypes, Objectification and Sexualization

**DOI:** 10.3390/ijerph20105770

**Published:** 2023-05-09

**Authors:** Fabrizio Santoniccolo, Tommaso Trombetta, Maria Noemi Paradiso, Luca Rollè

**Affiliations:** Department of Psychology, University of Turin, Via Verdi 10, 10124 Turin, Italy

**Keywords:** gender, media, representation, stereotypes, objectification, sexualization

## Abstract

Media representations play an important role in producing sociocultural pressures. Despite social and legal progress in civil rights, restrictive gender-based representations appear to be still very pervasive in some contexts. The article explores scientific research on the relationship between media representations and gender stereotypes, objectification and sexualization, focusing on their presence in the cultural context. Results show how stereotyping, objectifying and sexualizing representations appear to be still very common across a number of contexts. Exposure to stereotyping representations appears to strengthen beliefs in gender stereotypes and endorsement of gender role norms, as well as fostering sexism, harassment and violence in men and stifling career-related ambitions in women. Exposure to objectifying and sexualizing representations appears to be associated with the internalization of cultural ideals of appearance, endorsement of sexist attitudes and tolerance of abuse and body shame. In turn, factors associated with exposure to these representations have been linked to detrimental effects on physical and psychological well-being, such as eating disorder symptomatology, increased body surveillance and poorer body image quality of life. However, specificities in the pathways from exposure to detrimental effects on well-being are involved for certain populations that warrant further research.

## 1. Introduction

As a social category, gender is one of the earliest and most prominent ways people may learn to identify themselves and their peers, the use of gender-based labels becoming apparent in infants as early as 17 months into their life [1]. Similarly, the development of gender-based heuristics, inferences and rudimentary stereotypes becomes apparent as early as age three [2,3]. Approximately at this age, the development of a person’s gender identity begins [4]—that is, the process through which a person tends to identify as a man, as a woman or as a vast spectrum of other possibilities (i.e., gender non-conforming, agender, genderfluid, etc.). These processes continue steadily throughout individuals’ lives as they receive and elaborate information about women and men and what it means to belong to either category, drawing from direct and indirect observations, social contact, personal elaborations and cultural representations [5,6]. As a result, social and mental representations of gender are extremely widespread, especially as a strictly binary construct, and can be argued to be ubiquitous in individual and social contexts.

Among the many sources of influence on gender representations, media occupies an important space and its relevance can be assessed across many different phenomena [7,8,9,10,11]. The ubiquity of media, the chronicity of individuals’ exposure to it and its role in shaping beliefs, attitudes and expectations have made it the subject of scientific attention. In fact, several theories have attempted to explore the mechanisms and psychological processes in which media plays a role, including identity development [12,13,14], scripts and schemas [15], cultivation processes [16,17,18] and socialization processes [5,6].

The public interest in the topic of gender has seen a surge in the last 10 years, in part due to social and political movements pushing for gender equality across a number of aspects, including how gender is portrayed in media representations. In the academic field as well, publications mentioning gender in their title, abstract or keywords have more than doubled from 2012 to 2022 [19], while publications mentioning gender in media representations have registered an even more dramatic increase, tripling in number [20]. Additionally, the media landscape has had a significant shift in the last decade, with the surge in popularity and subsequent addition of social media websites and apps to most people’s mediatic engagement [21].

The importance of media use in gender-related aspects, such as beliefs, attitudes, or roles, has been extensively documented. As reported in a recent review of the literature [22], several meta-analyses [17,23,24] showed support for the effects of media use on gender beliefs, finding small but consistent effect sizes. These effects appear to have remained present over the decades [25].

Particular attention has been given to stereotypical, objectifying and sexualizing representations, as portrayals that paint a restrictive picture of the complexity of human psychology, also producing sociocultural pressures to conform to gender roles and body types.

Gender stereotypes can be defined as an extremely simplified concept of attitudes and behaviors considered normal and appropriate for men and women in a specific culture [26]. They usually span several different areas of people’s characteristics, such as physical appearance, personality traits, behaviors, social roles and occupations. Stereotypical beliefs about gender may be divided into descriptive (how one perceives a person of a certain gender to be; [27]), prescriptive (how one perceives a person of a certain gender should be and behave; [28,29]) or proscriptive (how one perceives a person of a certain gender should not be and behave; [28,29]). Their content varies on the individual’s culture of reference [30], but recurring themes have been observed in western culture, such as stereotypes revolving around communion, agency and competence [31]. Women have stereotypically been associated with traits revolving around communion (e.g., supportiveness, compassion, expression, warmth), while men have been more stereotypically associated with agency (e.g., ambition, assertiveness, competitiveness, action) or competence (e.g., skill, intelligence). Both men and women may experience social and economic penalties (backlash) if they appear to violate these stereotypes [29,32,33].

Objectification can be defined as the viewing or treatment of people as objects. Discussing ways in which people may be objectified, Nussbaum first explored seven dimensions: instrumentality (a tool to be employed for one’s purposes); denial of autonomy (lacking self-determination, or autonomy); inertness (lacking in agency or activity); fungibility (interchangeable with others of the same type); violability (with boundaries lacking integrity and permissible to break into); ownership (possible to own or trade); denial of subjectivity (the person’s feelings or experiences are seen as something that does not need to be considered) [34].

In its initial definition by Fredrickson and Roberts [35], objectification theory had been offered as a framework to understand how the pervasive sexual objectification of women’s bodies in the sociocultural context influenced their experiences and posed risks to their mental health—a phenomenon that was believed to have uniquely female connotations. In their model, the authors theorized that a cultural climate of sexual objectification would lead to the internalization of objectification (viewing oneself as a sexual and subordinate object), which would in turn lead to psychological consequences (e.g., body shame, anxiety) and mental health risks (e.g., eating disorders, depression). Due to the pervasiveness of the cultural climate, objectification may be difficult to detect or avoid, and objectification experiences may be perceived as normative.

Sexual objectification, in which a person is reduced to a sexual instrument, can be construed to be a subtype of objectification and, in turn, is often defined as one of the types of sexualization [36]. As previously discussed by Ward [37], it should be made clear that the mere presence of sexual content, which may be represented in a positive and healthy way, should not be conflated with sexualized or objectifying representations.

The American Psychological Association’s 2007 report defines sexualization as a series of conditions that stand apart from healthy sexuality, such as when a person’s value is perceived to come mainly from sexual appeal or behavior, when physical attractiveness is equated to sexual attractiveness, when a person is sexually objectified or when sexuality is inappropriately imposed on a person [36]. Sexualization may involve several different contexts, such as personal, interpersonal, and cultural. Self-sexualization involves treating oneself as a sexual object [35]. Interpersonal contributions involve being treated as sexual objects by others, such as family or peers [38,39]. Finally, contributions by cultural norms, expectations and values play a part as well, including those spread by media representations [36]. After this initial definition, sexualization as a term has also been used by some authors (e.g., Zurbriggen & Roberts [40]) to refer to sexual objectification specifically, while others (e.g., Bigler and colleagues [41]) stand by the APA report’s broader meaning. In this section, we will explore scientific literature adopting the latter.

These portrayals have been hypothesized to lead to negative effects on people’s well-being on a mental and physical level, as well as bearing partial responsibility for several social issues, such as sexism, gender discrimination and harassment. However, the pathways that lead from an individual’s relationship with media to these detrimental effects can be complex. Furthermore, they seem to involve specificities for men and women, as well as for different sexual orientations. A wealth of publications has been produced on these themes and, to the authors’ knowledge, no recent review has attempted to synthesize their findings.

The present article aims to summarize the state of the art of research on stereotyping, sexualization and objectification in gender and media representations. A focus will be placed on the definitions of these concepts, the media where they occur, and verifying whether any changes over time are detectable or any specificities are present. The possible effects of these representations on people’s well-being will be explored as well.

A search of the literature was conducted on scientific search engines (APA PsycArticles, CINAHL Complete, Education Source, Family Studies Abstracts, Gender Studies Database, MEDLINE, Mental Measurements Yearbook, Sociology Source Ultimate, Violence & Abuse Abstracts, PUBMED, Scopus, Web of Science) to locate the most relevant contributions on the topic of media and gender representation, with a particular focus on stereotypes, objectification and sexualization, their presence in the media and their effects on well-being. Keywords were used to search for literature on the intersection of the main topics: media representation (e.g., media OR representation* OR portrayal*), gender (e.g., gender OR sex OR wom* OR m*n) and stereotypes, objectification and sexualization (e.g., stereotyp*, objectif*, sexualiz*). In some cases, additional keywords were used for the screening of studies on specific media (e.g., television, news, social media). When appropriate, further restrictions were used to screen for studies on effects or consequences (e.g., effect* OR impact* OR consequence* OR influence* OR outcome*). Inclusion criteria were the following: (a) academic articles (b) pertaining to the field of media representations (c) pertaining to gender stereotypes, objectification or sexualization. A dataset of 195 selected relevant papers was created. Thematic analysis was conducted following the guidelines developed by Braun and Clarke [42], in order to outline patterns of meaning across the reviewed studies. The process was organized into six phases: (1) familiarization with the data; (2) coding; (3) searching for themes; (4) reviewing themes; (5) defining and naming themes; and (6) writing up. After removing duplicates and excluding papers that did not meet the inclusion criteria, a total of 87 articles were included in the results of this review. The findings were discussed among researchers (LR, FS, MNP and TT) until unanimous consensus was reached.

## 2. Results

### 2.1. Stereotypical Portrayals

Gender stereotypes appear to be flexible and responsive to changes in the social environment: consensual beliefs about men’s and women’s attributes have evolved throughout the decades, reflecting changes in women’s participation in the labor force and higher education [31,43]. Perceptions of gender equality in competence and intelligence have sharply risen, and stereotypical perceptions of women show significant changes: perceptions of women’s competence and intelligence have surpassed those relative to men, while the communion aspect appears to have shifted toward being even more polarized on being typical of women. Other aspects, such as perceptions of agency being more typical of men, have remained stable [31].

Despite these changes, gender representation in the media appears to be frequently skewed toward men’s representation and prominently features gender stereotypes. On a global scale, news coverage appears to mostly feature men, especially when considering representation as expert voices, where women are still underrepresented (24%) despite a rise in coverage in the last 5 years [44]. Underrepresentation has also been reported in many regional and national contexts, but exact proportions vary significantly in the local context. Male representation has been reported to be greater in several studies, with male characters significantly outnumbering female characters [45], doing so in male-led and mixed-led shows but not in female-led shows [46] in children’s television programming—a key source of influence on gender representations. Similar results have been found regarding sports news, whose coverage overwhelmingly focuses on men athletes [47,48] and where women are seldom represented.

Several analyses of television programs have also shown how representations of men and women are very often consistent with gender stereotypes. Girls were often portrayed as focusing more on their appearance [45], as well as being judged for their appearance [49]. The same focus on aesthetics was found in sports news coverage, which was starkly different across genders, and tended to focus on women athletes’ appearance, featuring overly simplified descriptions (vs. technical language on coverage of men athletes) [48]. In addition, coverage of women athletes was more likely in sports perceived to be more feminine or gender-appropriate [47,48,50]. Similarly, women in videogames appear to be both underrepresented and less likely to be featured as playable characters, as well as being frequently stereotyped, appearing in the role of someone in need of rescuing, as love interests, or cute and innocent characters [51]. In advertising as well, gender stereotypes have often been used as a staple technique for creating relatability, but their use may lead to negative cross-gender effects in product marketing [52] while also possibly furthering social issues. Hust and colleagues found that in alcohol advertisements, belief in gender stereotypes was the most consistent predictor of intentions to sexually coerce, showing significant interaction effects with exposure to highly objectifying portrayals [53]. Representation in advertising prominently features gender stereotypes, such as depicting men in professional roles more often, while depicting women in non-working, recreational roles, especially in countries that show high gender inequality [54]. A recent analysis of print ads [55] confirmed that some stereotypes are still prominent and, in some cases, have shown a resurgence, such as portraying a woman as the queen of the home; the study also found representations of women in positions of empowerment are, however, showing a relative increase in frequency. Public support, combined with market logic, appears to be successfully pushing more progressive portrayals in this field [56].

Both skewed representation and the presence of stereotypes have been found to lead to several negative effects. Gender-unequal representation has been found to stifle political [57] and career [58] ambition, as well as foster organizational discrimination [59]. Heavy media use may further the belief in gender stereotypes and has been found to be linked to a stronger endorsement of traditional gender roles and norms [60], which in turn may be linked to a vast number of detrimental health effects. In women, adherence and internalization of traditional gender roles have been linked to greater symptoms of depression and anxiety, a higher likelihood of developing eating disorders, and lower self-esteem and self-efficacy [36,61,62,63]. In men as well, adherence to traditional masculine norms has been linked to negative mental health outcomes such as depression, psychological distress and substance abuse [64], while also increasing the perpetration of risky behaviors [65,66] and intimate partner violence [65,67].

### 2.2. Objectifying Portrayals

Non-sexual objectifying representations appear to have been studied relatively little. They have been found to be common in advertising, where women are often depicted as purely aesthetic models, motionless and decorative [68]. They may also include using a woman’s body as a supporting object for the advertised product, as a decorative object, as an ornament to draw attention to the ad, or as a prize to be won and associated with the consumption of the advertised product [55].

The vast majority of the literature has focused on the sexual objectification of women. This type of representation has been reported to be very common in a number of contexts and across different media [69], and several studies (see Calogero and colleagues’ or Roberts and colleagues’ review [69,70]) have found support for the original model’s pathway [35]. Following experimental models expanded on the original (e.g., Frederick and colleagues or Roberts and colleagues [69,71]), highlighting the role of factors such as the internalization of lean or muscular ideals of appearance, finding evidence for negative effects on well-being and mental health through the increase in self-objectification and the internalization of cultural ideals of appearance [71,72].

Sexual objectification also appears to be consistently linked to sexism. For both women and men, the perpetration of sexual objectification was significantly associated with hostile and benevolent sexism, as well as the enjoyment of sexualization [73]. Enjoyment of sexualization, in turn, has been found to be positively associated with hostile sexism in both men and women, positively associated with benevolent sexism in women and negatively in men [74].

Exposure to objectifying media in men has been found to increase the tendency to engage in sexual coercion and harassment, as well as increasing conformity to gender role norms [75]. Consistently with the finding that perpetration of objectification may be associated with a greater men’s proclivity for rape and sexual aggression [76], a study conducted by Hust and colleagues found that exposure to objectifying portrayals of women in alcohol advertising was also a moderator in the relationship between belief in gender stereotypes and intentions to sexually coerce. Specifically, participants who had a stronger belief in gender stereotypes reported stronger intentions to sexually coerce when exposed to slightly objectifying images of women. Highly objectifying images did not yield the same increase—a result interpreted by the authors to mean that highly objectified women were perceived as sexually available and as such less likely to need coercion, while slightly objectified women could be perceived as more likely to need coercion [53].

Research on objectification has primarily focused on women, in part due to numerous studies suggesting that women are more subject to sexual objectification [73,77,78,79,80], as well as suffering the consequences of sexual objectification more often [81]. However, sexually objectifying portrayals seem to have a role in producing negative effects on men as well, although with partially different pathways. In men, findings about media appearance pressures on body image appear to be mixed. Previous meta-analyses found either a small average effect [82] or no significant effect [72]. A recent study found them to be significantly associated with higher body surveillance, poorer body image quality of life and lower satisfaction with appearance [71]. Another study, however, found differing relationships regarding sexual objectification: an association was found between experiences of sexual objectification and internalization of cultural standards of appearance, body shame and drive for muscularity, but was not found between experiences of sexual objectification and self-objectification or body surveillance [83]: in the same study, gender role conflict [84] was positively associated to the internalization of sociocultural standards of appearance, self-objectification, body shame and drive for muscularity, suggesting the possibility that different pathways may be involved in producing negative effects on men. Men with body-image concerns experiencing gender role conflict may also be less likely to engage in help-seeking behaviors [85,86]. This is possibly due to restrictive emotionality associated with the male gender role leading to more negative attitudes toward help-seeking, as found in a recent study by Nagai, [87], although this study finds no association with help-seeking behavior, conflicting with previous ones, and more research is needed.

Finally, specificities related to sexual orientation regarding media and objectification appear to be present. A set of recent studies by Frederick and colleagues found that gay men, lesbian women and bisexual people share with heterosexual people many of the pathways that lead from sociocultural pressures to internalization of thin/muscular ideals, higher body surveillance and a lower body image quality of life [71,88], leading the authors to conclude that these factors’ influence applies regardless of sexual orientation. However, their relationship with media and objectification may vary. Gay and bisexual men may face objectification in social media and dating apps rather than in mainstream media and may experience more objectification than heterosexual men [89]. In Frederick and colleagues’ studies, gay men reported greater media pressures, body surveillance, thin-ideal internalization, and self-objectification compared to heterosexual men; moreover, bisexual men appeared to be more susceptible to ideal internalization, displaying stronger paths from media appearance pressures to muscular-ideal internalization compared to heterosexual men; lesbian women, instead, demonstrated weaker relationships between media pressures and body image outcomes [71,88]. Consistently with previous studies suggesting a heightened susceptibility to social pressures [90], bisexual women appeared to be more susceptible to media pressures relative to other groups [88]. Another recent study of lesbian and bisexual women supported previous evidence for the pathway from the internalization of cultural appearance standards to body surveillance, body shame and eating disorder symptoms; however, it found no significant connection between experiences of objectification and eating disorder symptoms [91].

### 2.3. Sexualized Portrayals

Several studies have found sexualizing media representations to be commonplace across a number of different media contents and across different target demographics (i.e., children, adolescents or adults) and genres. Reports of common sexualized representations of women are found in contexts such as television programs [92], movies [93,94,95,96], music videos [97,98], advertising [54,55], videogames [51,99,100], or magazines [101].

Exposure to sexualized media has been theorized to be an exogenous risk factor in the internalization of sexualized beliefs about women [41], as well as one of the pathways to the internalization of cultural appearance ideals [102]. Daily exposition to sexualized media content has been consistently linked to a number of negative effects. Specifically, it has been found to lead to higher levels of body dissatisfaction and distorted attitudes about eating through the internalization of cultural body ideals (e.g., lean or muscular) in both men and women [71]. It has also been associated with a higher chance of supporting sexist beliefs in boys [103], and of tolerance toward sexual violence in men [104]. Furthermore, exposure to sexualized images has been linked to a higher tolerance of sexual harassment and rape myth acceptance [76]. Exposure to reality TV programs consistently predicted self-sexualization for both women and men, while music videos did so for men only [103]. Internalized sexualization, in turn, has been linked to a stronger endorsement of sexist attitudes and acceptance of rape myths [105], while also being linked to higher levels of body surveillance and body shame in girls [106]. Internalization of media standards of appearance has been linked to body surveillance in both men and women, as well as body surveillance of the partner in men [107].

As a medium, videogames have been studied relatively little and have produced less definite results. This medium can offer the unique dynamic of embodiment in a virtual avatar, which has been hypothesized to be able to lead to a shift in self-perception (the “Proteus effect”, as formulated by Yee & Bailenson, [108]). While some studies have partially confirmed this effect, showing that exposure to sexualized videogame representations can increase self-objectification [109,110,111], others [112] have not found the same relationship. Furthermore, while a study has found an association between sexualized representations in videogames, tolerance of sexual abuse of women and rape myth acceptance [113], and in another, it was linked to a decreased real-life belief in women’s competence [114], a recent meta-analysis [115] found no effect of the presence of sexualized content on well-being, sexism or misogyny.

Research on social media has also shown some specificities. Social media offers the unique dynamic of being able to post and disseminate one’s own content and almost always includes built-in mechanisms for user-generated feedback (e.g., likes), as well as often being populated by one’s peers, friends and family rather than strangers. Sites focusing on image- or video-based content (e.g., Instagram, TikTok) may be more prone to eliciting social comparison and fostering the internalization of cultural appearance ideals, resulting in more associations to negative body image when compared to others that have the same capabilities but offer text-based content as well (e.g., Facebook) [116]. Social media appears to foster social comparison, which may increase appearance-based concerns [117]. Consistently with previous research, exposure to sexualized beauty ideals on social media appeared to be associated with lower body satisfaction; exposure to more diverse standards of appearance, instead, was associated with increased body satisfaction and positive mood, regardless of image sexualization [116,118].

## 3. Discussion

### 3.1. Critical Discussion of Evidence

The reviewed evidence (summarized in Table 1) points to the wide-ranging harmful effects of stereotyping, objectifying and sexualizing media portrayals, which are reported to be still both common and pervasive. The links to possible harms have also been well documented, with a few exceptions.

These representations, especially but not exclusively pertaining to women, have been under social scrutiny following women’s rights movements and activism [119] and can be perceived to be politically incorrect and undesirable, bringing an aspect of social desirability into the frame. Positive attitudes toward gender equality also appear to be at an all-time high across the western world [120,121], a change that has doubtlessly contributed to socio-cultural pressure to reduce harmful representations. Some media contexts (e.g., advertising and television) seem to have begun reflecting this change regarding stereotypes, attempting to either avoid harmful representations or push more progressive portrayals. However, these significant changes in stereotypes (e.g., regarding competence) have not necessarily been reflected in women’s lives, such as their participation in the labor force, leadership or decision-making [31,122,123]. Objectifying or sexualizing representations do not seem to be drastically reduced in prevalence. Certainly, many influences other than media representations are in play in this regard, but their effect on well-being has been found to be pervasive and consistent. Despite widespread positive attitudes toward gender equality, the persistence of stereotypical, objectifying and sexualizing representations may hint at the continued existence of an entrenched sexist culture which can translate into biases, discrimination and harm.

Despite some conflicting findings, the literature also hints at the existence of differences in how media pressures appear to affect men and women, as well as gay, lesbian and bisexual people. These may point to the possibility of some factors (e.g., objectification) playing a different role across different people in the examined pathways, an aspect that warrants caution when considering possible interventions and clinical implications. In some cases, the same relationship between exposure to media and well-being may exist, but it may follow different pathways from distal risk factors to proximal risk factors, as in the case of gender role conflict for men or body shame for lesbian and bisexual women. However, more research is needed to explore these recent findings.

Different media also appear to feature specificities for which more research is needed, such as videogames and social media. The more interactive experiences offered by these media may play an important role in determining their effects, and the type of social media needs to be taken into consideration as well (image- or video-based vs. text-based). Moreover, the experiences of exposure may not necessarily be homogenous, due to the presence of algorithms that determine what content is being shown in the case of social media, and due to the possibility of player interaction and avatar embodiment in the case of videogames.

Past findings [37,69] about links with other social issues such as sexism, harassment and violence appear to still be relevant [67,73,103,105]. The increases in both tolerance and prevalence of sexist and abusive attitudes resulting from exposure to problematic media representations impact the cultural climate in which these phenomena take place. Consequently, victims of discrimination and abuse living in a cultural climate more tolerant of sexist and abusive attitudes may experience lower social support, have a decreased chance of help-seeking and adopt restrictive definitions for what counts as discrimination and abuse, indirectly furthering gender inequalities.

Exploring ways of reducing risks to health, several authors [22,41,75] have discussed media literacy interventions—that is, interventions focused on teaching critical engagement with media—as a possible way of reducing the negative effects of problematic media portrayals. As reported in McLean and colleagues’ systematic review [124], these interventions have been previously shown to be effective at increasing media literacy, while also improving body-related outcomes such as body satisfaction in boys [125], internalization of the thinness ideal in girls [125], body size acceptance in girls [126] and drive for thinness in girls and boys [127]. More recently, they were also shown to be effective at reducing stereotypical gender role attitudes [128], as well as fostering unfavorable attitudes toward stereotypical portrayals and lack of realism [129]. Development and promotion of these interventions should be considered when attempting to reduce negative media-related influences on body image. It should be noted, however, that McLean and colleagues’ review found no effect of media literacy interventions on eating disorder symptomatology [124], which warrants more careful interventions.

Furthermore, both internal (e.g., new entrants’ attitudes in interpersonal or organizational contexts) and external (e.g., pressure from public opinion) sociocultural pressures appear to have a strong influence in reducing harmful representations [55,56]. Critically examining these representations when they appear, as well as voicing concerns toward examples of possibly harmful representations, may promote more healthy representations in media. As documented by some studies, the promotion of diverse body representations in media may also be effective in reducing negative effects [70,118].

### 3.2. Limitations

The current review synthesizes the latest evidence on stereotyping, objectifying and sexualizing media representations. However, limitations in its methodology are present and should be taken into consideration. It is not a systematic review and may not be construed to be a complete investigation of all the available evidence. Only articles written in the English language have been considered, which may have excluded potentially interesting findings written in other languages. Furthermore, it is not a meta-analysis, and as such cannot be used to draw statistical conclusions about the surveyed phenomena.

### 3.3. Future Directions

While this perception is limited by the non-systematic approach of the review, to what we know, very few studies appear to be available on the relationship between media representation and non-sexual objectification, which may provide interesting directions to explore in relation to autonomy, violability or subjectivity, as was attempted in the context of work and organizations [130].

More cross-cultural studies (e.g., Tartaglia & Rollero [54]) would also prove useful in exploring differences between cultural contexts, as well as the weight of different sociocultural factors in the relationship between media representation and gender.

More studies focusing on relatively new media (e.g., social media, videogames) would possibly help clear up some of the identified discrepancies and explore new directions for the field that take advantage of their interactivity. This is particularly true for niche but growing media such as virtual reality, in which the perception of embodiment in an avatar with different physical features than one’s own could prove to be important in sexualization and objectification. Only preliminary evidence [131] has been produced on the topic.

Studies to further explore the relationship between media representations, gender and sexual orientation would also be beneficial. As already highlighted by Frederick and colleagues [132], gay, lesbian and bisexual people may deal with a significantly different set of appearance norms and expectations [133], and face minority-related stresses [134] that can increase susceptibility to poorer body image and disordered eating [135,136]. Additionally, none of the reviewed studies had a particular focus on trans people, who may have different experiences relating to media and body image, as suggested by the differences in pathways found in a recent study [137]. Sexual orientation and gender identity should be kept into consideration when investigating these relationships, as their specificities may shed light on the different ways societal expectations influence the well-being of sexual minorities.

The examined literature on the topic also appears to feature specificities that need to be taken into account. As previously reported by Ward [37], the vast majority of the studies continue to be conducted in the United States, often on undergraduates, which limits the generalizability of the results to the global population. Given the abundance and complexity of the constructs, more studies examining the pathways from media exposure to well-being using methodologies such as path analysis and structural equation modeling may help clarify some of the discrepancies found in the literature about the same relationships.

Finally, as previously reported by many authors [37,69,138], sexualization, self-sexualization, objectification and self-objectification are sometimes either treated as synonymous or used with different definitions and criteria, which may add a layer of misdirection to studies on the subject. Given the divergences in the use of terminology, clearly stating one’s working definition of sexualization or objectification would possibly benefit academic clarity on the subject.

## 4. Conclusions

Consistent empirical evidence highlights the importance of media representations as a key part of sociocultural influences that may have consequences on well-being. Despite some notable progress, harmful representations with well-researched links to detrimental effects are still common across a number of different media. Exposure to stereotyping, objectifying and sexualized representations appears to consistently be linked to negative consequences on physical and mental health, as well as fostering sexism, violence and gender inequity. On a clinical level, interventions dealing with body image and body satisfaction should keep their influence into account. The promotion of institutional and organizational interventions, as well as policies aimed at reducing their influence, could also prove to be a protective factor against physical and mental health risks.

## Figures and Tables

**Table 1:** Summary of findings.

	Gender Stereotypes	Objectification	Sexualization
**Frequency of portrayal**	Common	Common	Common
**Exposure** **effects**	**Both genders**: Higher belief in gender stereotypes; endorsement of traditional gender roles. **Women**: reduction of political and career-related ambition; organizational discrimination.	**Both genders**: Internalization of cultural ideals of appearance; increase in self-objectification; hostile and benevolent sexism; enjoyment of sexualization. **Men**: proclivity for sexual coercion (moderator); conformity to gender role norms.	**Both genders**: Internalization of cultural ideals of appearance; self-sexualization. **Men**: higher support of sexist beliefs (boys); tolerance toward sexual violence.
**Indirect** **effects**	**Women**: Symptoms of depression and anxiety; higher likelihood of eating disorders; lower self-esteem and self-efficacy. **Men**: symptoms of depression, psychological distress; higher proclivity for sexual coercion; substance abuse, increased perpetration of risky behaviors, intimate partner violence.	**Both genders**: higher likelihood of eating disorders and disordered eating behaviors	**Both genders**: higher levels of body dissatisfaction; body surveillance; distorted attitudes about eating; higher endorsement of sexist attitudes; acceptance of rape myths. **Women**: body shame (girls). **Men**: body surveillance of the partner.
**Conflicting** **research**	–	**Men**: media appearance pressures on body image	Effects of exposure to videogames
**Understudied areas**	Virtual reality	Non-sexual portrayals; specificities of sexual minorities; virtual reality	Specificities of videogames; specificities of sexual minorities; virtual reality
